# Diet satisfaction and associated factors among adult surgical orthopaedic inpatients at a teaching hospital in Lusaka province, Zambia; a hospital-based cross-sectional study

**DOI:** 10.1186/s40795-019-0288-5

**Published:** 2019-04-01

**Authors:** Nixon Miyoba, Irene Ogada

**Affiliations:** 1 Department of Food, Nutrition and Dietetics, Kenyatta University, Nairobi, Kenya; 2 Kitwe Teaching Hospital, Nutrition and Dietetics Unit, P.O. Box 20969, Kitwe, Zambia; 3 Saint Francis Xavier University, Antigonish, Canada

**Keywords:** Diet satisfaction, Food service, Lusaka province, Teaching hospital, Surgical orthopaedic inpatients

## Abstract

**Background:**

Poor quality of food services in hospital contributes to low diet satisfaction among inpatients in both developed and developing countries. However, there is paucity of literature on diet satisfaction in health care facilities in the sub-Saharan region and in particular Zambia. Therefore, this study sought to assess levels of diet satisfaction among adult surgical orthopaedic inpatients at a teaching hospital in Lusaka province, Zambia.

**Methods:**

A hospital-based cross-sectional study was conducted over a period of three months. Comprehensive sampling was used to select 98 study participants. A researcher-administered questionnaire adapted from a similar study was used to collect data. The instrument used in this study had 9 aspects of satisfaction. Descriptive statistics such as frequencies, percentages, means and standard deviations were used to analyze the data. Chi-square test was used to test for associations between categorical data. A *p*-value of less than 0.05 was considered to be statistically significant.

**Results:**

In this study, 64.3% of surgical orthopaedic inpatients were not satisfied with overall quality of hospital food. In addition, 76.5, 96.9, 65.3 and 71.4% of the patients were not satisfied with type, variety, appearance and taste of hospital food respectively. However, patients who were satisfied with portion size, temperature and time of meal distribution were 67.3, 94.9 and 56.1% respectively. There was no significant association between variables of age, sex, education level, marital status, monthly income, days in hospital and overall satisfaction (*p* > 0.05).

**Conclusion:**

Low diet satisfaction is a global problem associated with poor quality of hospital meals. Although the majority of surgical orthopaedic inpatients were not satisfied with more than half of the dimensions of diet satisfaction, they were satisfied with aspects of portion size, temperature and time of meal distribution. Therefore, an assessment of diet satisfaction can inform hospital administrators and policy makers on the deficiencies in hospital diets and thereby help improve quality of meals.

**Electronic supplementary material:**

The online version of this article (10.1186/s40795-019-0288-5) contains supplementary material, which is available to authorized users.

## Background

Orthopaedic injuries are common globally, affecting hundreds of millions of people [1]. According to literature, the global burden of orthopaedic injuries will continue to grow. In 1998 and 2001, injuries accounted for 16 and 11% respectively of the world’s disease burden [2]. Literature indicates that the prevalence of general disability associated with orthopaedic incidents is highest in sub-Saharan Africa [3, 4]. The most prevalent orthopaedic conditions are those associated with fractures, dislocations and sprains [4]. Most orthopaedic victims seek medical treatment in hospitals, where they may be admitted and become entirely or partially dependent on hospital meals. The goal of any service institution is to satisfy the needs of its’ clients. When patients visit hospitals, they have expectations that can either be met or not [5]. In both developed and developing countries, quality of food in hospitals is a common problem that contributes to low diet satisfaction [6]. One of the major challenges facing hospital administrators is the provision of quality meals that meet the expectations of patients [7].

Patients who are dissatisfied with hospital meals are likely to develop malnutrition due to reduced food intake [8–10]. However, improvement in the quality of food services is one of the strategies that can contribute to quick recovery of inpatients [11]. Moreover, improved food services have the capacity to influence nutritional status, which in turn is directly related with healing outcomes [12, 13]. According to published literature, quality of food in hospitals is also significantly associated with overall patient satisfaction [14]. There are multiple factors that contribute to satisfaction and one important predictor is the patients’ relationship with food [15]. Studies report that patient satisfaction is one of the recognized indicators that can be used to assess the quality of hospital food [6, 11, 12].

A study done in Saudi Arabia reported that 78.8% of the patients were satisfied with quality of hospital food [6]. A similar study conducted in Malaysia reported that although only 6.3% of the patients were not happy with hospital food, more than 50% of them supplemented hospital meals with outside food [8]. Despite most of the patients being satisfied with hospital food, a study in India indicated that 38.3% of them were dissatisfied with a monotonous diet [11]. However, results of a study in Greece suggested that most of the patients were satisfied with variety and quality of hospital meals [13]. In spite of the fact that most studies elsewhere report that patients are satisfied with overall food services, levels of satisfaction with specific diet elements may vary from country to country. There is paucity of information in developing countries such as Zambia on diet satisfaction of inpatients. Therefore, the purpose of this study was to evaluate diet satisfaction among adult surgical orthopaedic patients and establish associated factors. Results of this study may help identify gaps in meal provision and thereby contibute to formulation of policy aimed at improving the quality of hospital meals.

## Methods

### Study design, setting and location

This study adopted a cross-sectional analytical study design in data collection, analysis and presentation. The design was appropriate for this study to test for associations between demographic characteristics and satisfaction [16]. The study was conducted at a teaching hospital with a 1655 bed capacity and offers various specialist services to the public and is the primary trauma center in Lusaka [17, 18]. It was carried out from June to October 2016. The bed occupancy rate at the teaching hospital is about 85%, while the average length of stay in hospital for patients ranges from 5 to 6 days. Some wards at the hospital are designated low cost, while others are high cost [19]. Patients in low cost wards receive free medical services compared to those in high cost wards who pay for health services [19]. Low cost wards are subsidized by the government [19]. Ethical approval for this study was obtained from Eres Converge in Zambia.

### Study population

The study targeted adult orthopaedic patients admitted in low cost surgical wards on standard hospital diet. Eligible to participate in this study were adult surgical orthopaedic inpatients, 18–64 years, able to communicate and on standard hospital diet ≥3 days. This period of time is long enough for patients to form an opinion about hospital foodservices. The study’s exclusion criteria were paediatrics, cognitively impaired patients, patients with critical conditions and those who declined to participate.

According to the food service staff at the teaching hospital, orthopaedic patients like other patients in low cost wards are served three main meals comprised of breakfast, lunch and supper. The hospital does not provide any snacks to orthopaedic patients. With regards the food service system used to serve patients in low cost wards, the hospital uses a centralized system whereby food is prepared in the main kitchen by cooks. Once the food is ready, it is held hot in covered buckets and using trolleys, delivered fresh, plated on the ward and served as soon as possible by waiters. However, the food service system in high cost wards is different in the sense that food is prepared on demand for patients who can pay for food services. Furthermore, patients in high cost wards can choose food from a paper menu provided and food is plated right in the kitchen prior to distribution by waiters. In low cost wards, patients are offered soya porridge at breakfast and beans is served almost on a daily basis for lunch or supper. Beef is the only animal product that is provided by the hospital and is supplied irregularly because the hospital lacks a fixed menu. Soya pieces are served fortnightly to orthopaedic patients and that the only vegetable supplied by the hospital is cabbage and is served once or twice per week. Nshima (cooked maize meal cereal) is the only cereal that is served to patients at lunch and supper. The hospital does not offer any fruits to orthopaedic patients.

### Sample size and sampling procedure

The teaching hospital was purposively sampled out of the four main referral hospitals in the country because it is the primary trauma center with a relatively higher number of adult surgical orthopaedic patients [18]. Low cost surgical wards (male and female) were also purposively sampled because that is where orthopaedic patients who eat regular hospital meals are admitted. Participants were drawn from all low cost (three male and two female) surgical wards. The nurses-in-charge of each of the selected low cost surgical wards were requested for the admission records from which a sampling frame was generated [20]. Comprehensive sampling was used to include all participants who satisfied the inclusion criteria and consented to participate in the study [21, 22].

### Research instruments

The researcher-administered questionnaire and data collection procedures were pre-tested at a similar referral hospital in Zambia (Additional file 1). The pre-test was done on a selected sample of 10 adult surgical orthopaedic patients with conditions similar to those of the main study. The pre-test participants were not included in the main study. The procedures employed in pre-testing the instruments were identical to those used in the main study. Validity was ensured by use of already validated tool from a similar study [6]. The test-retest method was used to determine the reliability of the instruments during pretest. Data was collected twice during the pretest at an interval of 2 days from 10 participants. A test re-test correlation coefficient of 0.72 (CI: 0.61–0.82) was computed from the two sets of data and found to be adequate [23]. In order to avoid bias in patient behaviour, attending doctors, nurses and support staff were not informed about the study except in unavoidable situations [24].

### Recruitment and training of two research assistants

Recruitment of research assistants was done competitively on the basis of: possession of a diploma in nutrition, conversant with English and two other languages widely spoken in Lusaka (Bemba and Nyanja), prior experience in research and a resident of Lusaka. Successful candidates were trained by the researcher for four days with emphasis on the following thematic areas; research ethics, study purpose and objectives, interviewing techniques, and correct recording of data in the questionnaires. The training was conducted through role plays, lectures, demonstrations and a pre-test.

### Data collection procedures

The instrument used in this study had 9 dimensions of diet satisfaction. They were type, portion size, variety, taste, appearance, time of food distribution, temperature, overall quality, and attitude of staff serving hospital food. Patients were requested to indicate their level of satisfaction by selecting responses on a five-point Likert rating scale. The points associated with each scale were as follows; 1 = very dissatisfied, 2 = dissatisfied, 3 = fairly satisfied, 4 = satisfied, 5 = very satisfied. Patients who choose “very dissatisfied” and “dissatisfied” were considered dissatisfied, while those who selected “fairly satisfied”, “satisfied” and “very satisfied” were regarded satisfied. Satisfied participants were associated with a mean score of 3 and above, while dissatisfied patients had mean scores of 1 and 2. The instrument also contained questions on socio-demographic characteristics of the patients. The questionnaire was administered for 30–35 min.

### Statistical analyses

Statistical Package for Social Sciences (SPSS) version 21.0 was used to analyze data. Descriptive statistics in terms of means, frequencies, percentages, and standard deviations were generated. Chi-square test was used to determine associations between variables such as age categories, length of stay in hospital, education level, income categories and satisfaction. Test of quantitative variables for normal distribution was done using the Kolmogorov-Smirnoff test. A *p*-value of less than 0.05 was considered statistically significant.

## Results

### Socio-demographic characteristics of the patients

A total of 104 patients were eligible to participate, 100 consented to be interviewed, while 4 declined. Out of those who consented, data was missing for 2 patients. Therefore, a total of 98 patients had fully completed data and constituted the sample size. The mean age of the orthopaedic inpatients was 36.4 ± 9.44 years, with the majority being 30–39 years of age (39.8%). The mean number of days spent in hospital was 17.33 ± 10.91 (Table 1). Majority of the orthopaedic patients had attained primary education (45.9%), while 39.8% had attained secondary education. Most of the study participants (62.2%) had fractures, while 24.9% had soft tissue injuries.

**Table 1:** Socio-demographic characteristics of adult surgical orthopaedic in-patients

Variable	*N* = 98	%
Age		
≤ 30 years	27	27.6
31–50	61	62.2
> 50	10	10.2
Sex		
Male	79	80.6
Female	19	19.4
Education level		
No formal education	9	9.2
Primary	45	45.9
Secondary	39	39.8
Tertiary	5	5.1
Marital status		
Married	62	63.3
Not married	36	36.7
Length of stay		
3–7 days	21	21.4
8–16 days	36	36.7
> 16 days	41	41.8
Occupation		
Formal	14	14.3
Informal	68	69.4
Unemployed	16	16.3
Monthly income		
≤ K1000	39	39.8
K1001 – K2000	33	33.7
K2001 – K3000	13	13.3
K3001 – K4000	2	2
≥ K4001	11	11.2
Common orthopaedic injuries		
Fractures	45	45.9
Dislocations	18	18.4
Soft tissue injuries	24	24.5
Malformed bones	6	6.1
others	5	5.1

K stands for Zambian Kwacha

### Diet satisfaction of the study participants

Majority of the orthopaedic patients were satisfied (67.3%) with aspects of portion size, temperature of hospital food (94.9%) and slightly over half (56.1%) were satisfied with time of meal distribution (Table 2). Only 34.7% of the patients were satisfied with appearance of hospital food. Most of the patients interviewed reported to be dissatisfied with aspect of type of hospital food, variety and taste (76.5, 96.9 and 71.4% respectively) (Table 3). When patients were asked on overall quality and attitude of staff serving hospital food, 64.3 and 68.4% respectively reported that they were not satisfied.

**Table 2:** Satisfaction of participants with different aspects of hospital food

Aspect of food	N = 98						
	Satisfied				Dissatisfied		
	Very satisfied	Satisfied	Fairly satisfied	Total n(%)	Dissatisfied	Very dissatisfied	Total n(%)
Portion size	6	28	32	66 (67.3)	28	4	32 (32.7)
Temperature of hospital food	8	50	35	93 (94.9)	4	1	5 (5.1)
Time of meal distribution	5	20	30	55 (56.1)	35	8	43 (43.9)
Type of hospital food	0	10	13	23 (23.5)	45	30	75 (76.5)
Variety of hospital food	0	2	1	3 (3.1)	33	62	95 (96.9)
Taste of hospital food	0	20	8	28 (28.6)	62	8	70 (71.4)
Appearance of hospital food	0	19	15	34 (34.7)	55	9	64 (65.3)
Overall quality of hospital food	0	25	10	35 (35.7)	58	5	63 (64.3)
Attitude of staff serving food	0	20	11	31 (31.6)	53	14	67 (68.4)

**Table 3:** Patients’ satisfaction with hospital food by general characteristics

Variable	Satisfied		Not satisfied		P-value
	*N* = 35	%	*N* = 63	%	
Age					
< 30 years	12	34.3	15	23.8	0.250
31–50	18	51.4	43	68.3	
> 50	5	14.3	5	7.9	
Sex					
Male	27	77.1	52	82.5	0.517
Female	8	22.9	11	17.5	
Education					
No formal education	2	5.7	7	11.1	0.827
Primary	17	48.6	28	44.4	
Secondary	14	40	25	39.7	
Tertiary	2	5.7	3	4.8	
Marital status					
Married	20	57.1	42	66.7	0.349
Not married	5	429	21	33.3	
Length of stay					
3–7 days	9	28.6	11	17.5	0.433
8–16 days	7	34.3	24	38.1	
> 16 days	15	37.1	28	44.4	

### Diet satisfaction by general characteristics of patients

Results of chi-square test revealed no significant association between variables of age, sex, education level, marital status, monthly income, days in hospital and overall satisfaction (*p*>0.05).

## Discussion

Results of this study suggest that most of the participants were not satisfied with the overall quality of hospital meals. This is in contrast to the findings of a study conducted in India, which reported that majority of the patients were satisfied with hospital food [25]. However, study conducted in Kenya found that majority of the respondents rated food services as average [25]. Other studies conducted in Saudi Arabia and Egypt have reported that more than half of patients interviewed were satisfied with hospital food [6, 26]. Possible reasons for the variation in satisfaction is that each hospital offers a unique menu, the target population, methodological influences and individual values of patients may vary [6, 24]. Further, unlike our study, the studies done in Kenya and Egypt did not target patients with one medical condition [6].

The role played by the Dietetics and Catering Departments in the provision of therapeutic and regular diets to inpatients is very important [27]. This is because the care and recovery of patients is significantly influenced by sound dietary and meal services [24]. It is important for dietitians and nutritionists to be concerned not only with nutrient intake but also other aspects of hospital food that may affect dietary adequacy of inpatients. An important aspect of food services that is worth investigating is diet satisfaction. Satisfaction is a valid measure used to assess quality of service delivery in hospitals [6].

One major finding of the current study is that nearly all (96.9%) patients were dissatisfied with the variety of food at the hospital. This finding is supported by the report from a relevant foodservice officer who disclosed that the major challenge faced by the catering department is lack of variety in the hospital menu. A similar study in a government hospital in India reported that the major aspect attributed to dissatisfaction was monotony of hospital food [7]. Another study done in Bangladesh reported that meals provided to the hospital were not wholesome [10]. However, our finding is in contrast to those of a study in Greece that reported that most of the patients were pleased with the variety of hospital meals [13]. The possible explanation for lack of variety in hospital menus is that funding to the catering department may differ based on a country’s hospital food policies. In addition, variation in hospital menus may be influenced by the importance hospital managers attach to patients’ nutritional status and its impact on clinical outcomes.

This study found that patients were happy with aspects of portion size, temperature and time of meal distribution. The findings of our study are supported by a study in India which indicated that patients were pleased with portion size and time of meal distribution [24]. A similar study in Saudi Arabia suggested that majority of the patients were satisfied with amount, temperature and time of meal distribution [6]. However, a study done in Greece reported that patients were not happy with the temperature at which food is served [13]. Variations in satisfaction on variables of temperature and time of meal distribution maybe explained by the type of food service system available in a hospital. For example, to assure that food is hot, the system used at the teaching hospital is to serve it as soon as possible while the food is still covered in buckets.

Findings of the current study indicate that there was no significant association between patient specific characteristics such as age, sex, length of hospital stay and overall satisfaction (*p* > 0.05). Similar to our study results, a study in Saudi Arabia reported age and sex as insignificant variables affecting patient satisfaction [6]. A study in Kenya suggested that age was not a predictor of patient satisfaction [28]. On the other hand, studies in Sri Lanka and Kuwait suggested that satisfaction was influenced by age [29, 30]. Studies that include younger persons and older persons in most cases report an association between age and overall satisfaction. Therefore, the composition of the study population may to some extent explain the differences in findings.

Improvement in the quality of food services is one of the strategies that can contribute to quick recovery of inpatients [11]. Moreover, improved food services have the capacity to influence nutritional status and length of stay of inpatients [12, 13]. Contrary to the results of the current study, a similar study reported a correlation between length of stay and overall satisfaction [11]. However, results of a study in a military hospital reported a significant negative relationship [29]. Results suggested that the longer patients stay in hospital, they become dissatisfied with the hospital environment [31]. The observation seems not to agree with our findings perhaps because of the sample size and specific patient group included.

A study in Kenya reported that education level influenced overall satisfaction with food services [28]. The study suggested that patients without formal education were more satisfied with food services than those with tertiary education [28]. Literature documents that patient satisfaction with hospital food is influenced by factors such as patient’s education and perceived need of nutrition care [32]. A study in India reported higher levels of satisfaction among patients with high income [29]. This was in contrast to the findings of a study in Saudi Arabia that documented low monthly income as being significantly associated with higher levels of satisfaction [6]. In tandem with the findings of another study in Kenya, our study found no significant association between education level, monthly income and diet satisfaction [26].

### Study limitations

There were some limitations in this study such as data was only collected from one hospital in Zambia. The use of a comprehensive sample helped to eliminate sampling errors by providing data on all the individuals in the population [21, 22]. Comprehensive sampling techniques have been used before in other studies to collect data from small populations to include all participants [22]. In spite of the weakness highlighted, this study provides useful baseline information which might be of value to future studies in the country.

## Conclusions

Although most of the patients may not have been satisfied with overall quality of hospital food, they were satisfied with aspects of portion size, temperature, and time of meal distribution. Therefore, the patients did not negatively evaluate all aspects of hospital food. In spite of the weaknesses highlighted, the findings of this study are helpful in resource-poor settings like Zambia to help identify gaps in the quality of hospital meals. In order for the hospital to met the expectations of orthopeadic patients, gaps in the food service system such as absence of a fixed menu and lack of certain food groups in the menu should be addressed. The findings of the study may influence policy direction to increase funding towards improvement of quality of regular meals and thus meet the expectations of hospitalized patients.

## Additional file

Additional file 1: Questionnaire. (DOC 40 kb)

## Abbreviations

CI: Confidence interval
SD: Standard deviation
SPSS: Statistical Package for Social Sciences
TDRC: Tropical Disease Research Center
USA: United States of America
UTH: University Teaching Hospital
WHO: World Health Organization
