# Supplementary material for: Diet satisfaction and associated factors among adult surgical orthopaedic inpatients at a teaching hospital in Lusaka province, Zambia; a hospital-based cross-sectional study
Source: BMC Nutr. 2019 Apr 1;5:25. doi: 10.1186/s40795-019-0288-5 (PMC7050853; doi:10.1186/s40795-019-0288-5)
Supplement: Supplementary file 1 — Questionnaire. (DOC 40 kb) [file 40795_2019_288_MOESM1_ESM.doc]

**Questionnaire**

**Administrative details**

Questionnaire Code No:_________________ Name of the interviewer: _________________

Code No: __________________ Date of interview....../…../……

Start time_________ End time____________ Questionnaire checked ……………………

Complete the following questions by circling the correct response and writing the appropriate response or code in the last column.

**Section A**: Socio-demographic characteristics of the patients

| **S/N** | **Variable** | **Response** |
| --- | --- | --- |
| 1A | Age | 1=≤30 years  2=31 – 50  3=>50 |
| 2A | Sex | 1=Male  2=Female |
| 3A | Education level | 1=No formal education  2=Primary  3=Secondary  4=Tertiary |
| 4A | Marital status | 1=Married  2=Not married |
| 5A | Length of stay | 1=3 - 7 days  2=8 - 16 days  3=> 16 days |
| 6A | Employment status | 1=Formal  2=Informal  3=Unemployed |
| 7A | Monthly income | 1=≤ K1000  2=K1001 – K2000  3=K2001 – K3000  4=K3001 – K4000  5=≥ K4001 |
| 8A | Type of orthopaedic condition | 1=Fractures  2=Dislocations  3=Soft tissue injuries  4=Malformed bones  5=Others |

**Section B**: Likert rating scale -Satisfaction with hospital food and food services. Circle the appropriate response to indicate to what extent are you satisfied with hospital food

| **S/N** | **Variables** | **Responses** |
| --- | --- | --- |
| 1B | Portion size | 1=Very satisfied  2=Satisfied  3=Fairly satisfied  4=Dissatisfied  5=Very dissatisfied |
| 2B | Temperature of hospital food | 1=Very satisfied  2=Satisfied  3=Fairly satisfied  4=Dissatisfied  5=Very dissatisfied |
| 3B | Time of meal distribution | 1=Very satisfied  2=Satisfied  3=Fairly satisfied  4=Dissatisfied  5=Very dissatisfied |
| 4B | Type of hospital food  Variety of hospital food | 1=Very satisfied  2=Satisfied  3=Fairly satisfied  4=Dissatisfied  5=Very dissatisfied |
| 5B | Taste of hospital food | 1=Very satisfied  2=Satisfied  3=Fairly satisfied  4=Dissatisfied  5=Very dissatisfied |
| 6B | Appearance of hospital food | 1=Very satisfied  2=Satisfied  3=Fairly satisfied  4=Dissatisfied  5=Very dissatisfied |
| 7B | Overall quality of hospital food | 1=Very satisfied  2=Satisfied  3=Fairly satisfied  4=Dissatisfied  5=Very dissatisfied |
| 8B | Attitude of staff serving food | 1=Very satisfied  2=Satisfied  3=Fairly satisfied  4=Dissatisfied  5=Very dissatisfied |

**THANK YOU FOR YOUR PARTICIPATION**
